# Prevalence of acute reactions to gluten contamination of the diet in children with celiac disease

**DOI:** 10.3389/fped.2025.1635944

**Published:** 2025-09-10

**Authors:** Dorina Pjetraj, Denise Damiani, Chiara Monachesi, Salima Ricci, Milena Ascani, Simona Gatti, Carlo Catassi, Elena Lionetti

**Affiliations:** ^1^ Department of Pediatrics, Marche Polytechnic University, Ancona, Italy; ^2^ Mucosal Immunology and Biology Research Center, Massachusetts General Hospital-Harvard Medical School, Boston, MA, United States

**Keywords:** celiac disease, gluten-free diet, gluten exposure, gastrointestinal symptoms, gluten contamination

## Abstract

**Background and aim:**

The prevalence and clinical spectrum of symptoms due to inadvertent gluten exposure in children with celiac disease (CeD) on a gluten-free diet (GFD) are not well defined. This study aimed to assess these acute reactions through an online survey.

**Methods:**

Parents of children with CeD treated with a GFD for at least 12 months completed an online questionnaire. The survey focused on symptoms occurring within 24 h of gluten-contaminated food ingestion.

**Results:**

Data were collected for 296 children. Acute reactions after unintentional gluten ingestion were reported in 98 cases (33.1%). The most common symptoms were abdominal pain (57.1%), diarrhea (42.9%), vomiting (31.6%), headache (12.2%), and fatigue (14.3%). Less frequent symptoms included nausea, constipation, urticaria, aphthous stomatitis, and arthropathy (each ∼5%–7%). In 86% of cases, symptoms appeared within 2–3 h. Gluten exposure most often occurred while dining out, especially in restaurants and school cafeterias.

**Conclusions:**

One-third of children with CeD on a GFD experience acute reactions to accidental gluten ingestion. These reactions typically arise rapidly and are dominated by gastrointestinal symptoms, aligning with reports from existing literature, where vomiting and nausea have been observed in 3%–46% of patients at the time of CeD diagnosis and in 13%–61% during gluten challenge.

## 1 Introduction

Celiac disease (CeD) is an autoimmune enteropathy characterized by a chronic inflammatory response to the ingestion of gluten, a protein found in wheat, rye, and barley (1, 2). The disease can cause a wide variety of symptoms, both gastrointestinal and extraintestinal, and is estimated to affect approximately 1%–2% of the global population (3–5).

The treatment of CeD is a strict, lifelong gluten-free diet (GFD) which allows the intestinal mucosa to heal and prevents long-term complications of the disease (6, 7). However, some patients continue to report persistent symptoms despite following a GFD, often caused by gluten contamination into the diet (8).

Adhering to a GFD, which eliminates a common dietary staple across many countries, poses significant challenges and can negatively impact patients' psychosocial well-being and quality of life, particularly during vulnerable periods like adolescence. The complete avoidance of gluten is difficult to achieve, as naturally gluten-free items like oats and lentils can be cross-contaminated during processing. Furthermore, gluten is a widely used ingredient added for its functional properties and can be found in unsuspected food products (9). Cross-sectional studies have found that up to 50% of individuals with CeD who follow a GFD report consuming gluten, either intentionally or unintentionally (10, 11). Incomplete adherence to a GFD is more prevalent among males, adolescents, and individuals with clinically silent CeD (12). This unintended gluten intake can trigger an immune response and the reappearance of gastrointestinal and other symptoms (13, 14). Symptoms of active CeD usually manifest gradually over weeks or months. However, after starting treatment with the GFD, acute reactions to gluten ingestion are frequently reported by patients. To date, the prevalence of symptomatic acute reactions following unintentional gluten ingestion while on a GFD has not been fully investigated, particularly in children.

The aim of this study was to assess the occurrence and characteristics of such symptoms through an online survey of a large cohort of patients with CeD.

## 2 Materials and methods

### 2.1 Study design

This is a cross-sectional study, with data collected through an online survey performed from March to July 2024 at a regional referral center for pediatric CeD (Ancona, Italy). Prior to participating in the survey, parents of children with CeD who had been followed for at least 1 year were provided comprehensive details about the study and required to give written informed consent. All respondents were informed about the study's objectives, data usage, privacy, anonymity, confidentiality, and the voluntary nature of their participation, including the right to withdraw. The survey took approximately 15 min to complete. Participants who reported experiencing symptoms were followed up by a trained dietitian to verify the accuracy of their responses. Individuals were classified as having a positive reaction if they met the following criteria: (a) consistent symptom patterns following gluten exposure on at least two separate occasions, (b) symptom onset within 24 h of consuming a gluten-containing meal, (c) complete resolution of symptoms within 72 h. The research was conducted in accordance with the ethical principles outlined in the Declaration of Helsinki and was approved by the Ethics Committee of Marche, section AOU delle Marche (Ancona, Italy, ID 220059, approved 8th August 2023).

### 2.2 Survey participants

This online survey involved children/adolescents (age <18 years old) with a confirmed CeD diagnosis according to the ESPGHAN guidelines (15). Participants were asked about the occurrence of symptoms that manifested within 24 h following a documented incident of unintentional gluten consumption. The list of symptoms included abdominal pain, diarrhea, vomiting, headache, fatigue, nausea, constipation, urticaria, aphthous stomatitis and arthropathy. In symptomatic patients the timing, duration and severity of symptoms, as well as the setting in which the contamination occurred, were asked.

### 2.3 Statistical analysis

Descriptive statistics were used to summarize the data. Categorical variables were expressed as frequencies and percentages and compared using the chi-square test or Fisher's exact test, as appropriate. Continuous variables were reported as median and interquartile range (IQR) and compared using the Mann–Whitney *U* test. Statistical significance was defined as a *p*-value < 0.05. All analyses were performed using the R software (version 4.3.3).

## 3 Results

### 3.1 Study population

A total of 422 eligible patients were invited to participate in the study, and 296 of them completed the online survey (70%). Of these, 113 participants reported experiencing symptoms after gluten ingestion. However, following a structured follow-up with a trained dietitian to verify the timing, pattern, and resolution of symptoms, 15 participants did not fulfill the predefined criteria for a clear positive reaction to inadvertent gluten exposure. Ultimately, 98 participants (33.1%) (herein defined as “symptomatic”) reported experiencing symptoms after consuming gluten-contaminated meals. Demographic and clinical characteristics of the study population are reported in Table 1.

**Table 1:** Comparative analysis of clinical and demographic features of symptomatic and asymptomatic celiac disease patients.

	Symptomatic (*n* = 98)	Symptomless (*n* = 198)	*p* value
Female, *n* (%)	65 (66%)	107 (54%)	**0** **.** **043**
Age, median (IQR), years	10 (7–12)	10 (7–13)	0.287
Age at diagnosis, median (IQR), years	5 (3–8)	7 (5–10)	**0** **.** **001**
Symptomatic at diagnosis, %	91%	74%	**0** **.** **026**
Diagnosis confirmation:			
Serology-based diagnosis (ESPGHAN guidelines) %	74%	68%	0.256
Biopsy-based %	26%	32%	
Age			
≤6 years (%)	25.5%	30.8%	0.327
7–12 (%)	52%	51.5%	0.930
13–18 (%)	22.5%	17.7%	0.344
Gluten ingestion to appearance of symptoms median lag time (IQR), minutes	60 (30–120)		

Bolded values indicate a statistically significant result (*p*-values <0.05).

Female participants were more likely to report experiencing symptoms (66%) after the ingestion of contaminating gluten. The group of patients who reported symptoms differed from the asymptomatic group in the timing of their CeD diagnosis and symptoms at diagnosis. No significant differences were found among different age groups.

### 3.2 Symptom profile

**Figure 1:**
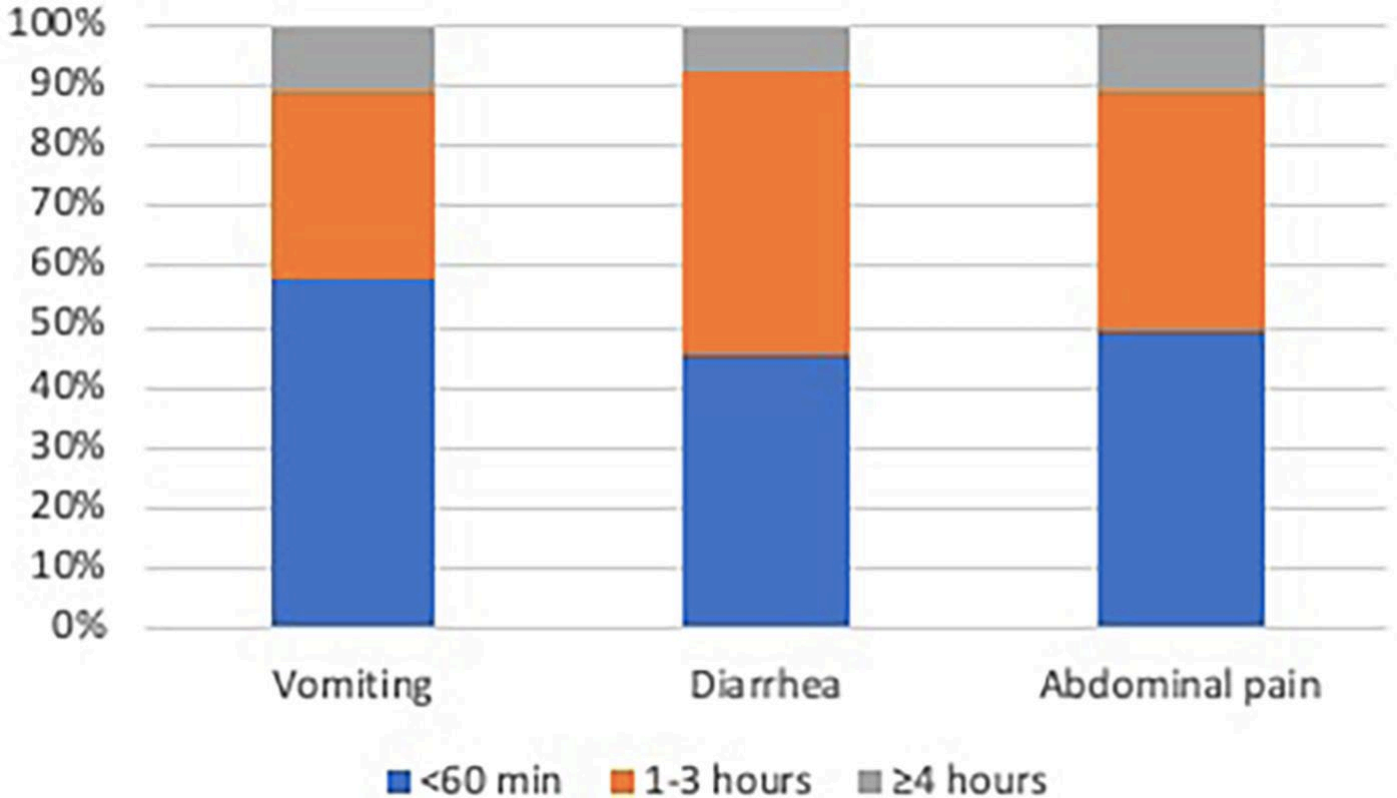
Cumulative frequency of the onset timing of the three most commonly reported symptoms (vomiting, diarrhea, abdominal pain).

The most prevalent symptoms were abdominal pain (56/98, 57.1%), followed by diarrhea (42/98, 42.9%), vomiting (31/98, 31.6%), headache (12/98, 12.2%), fatigue (14/98, 14.2%), nausea (7/98, 7.1%), constipation (7/98, 7.1%), urticaria (7/98, 7.1%), aphthous stomatitis (5/98, 5.1%) and arthropathy (5/98, 5.1%). The majority (>90%) of patients who experienced adverse effects reported that the symptoms emerged within a brief timeframe of less than 3 h (Figure 1).

Among the symptomatic participants, 63% (95% CI: 47.55–76.79) reported experiencing the same symptoms they had prior to CeD diagnosis and the initiation of the GFD. The most frequently reported new symptoms were gastrointestinal, such as diarrhea (58.8%), abdominal pain (47.0%), and vomiting (35.3%). Notably, 13 of the 98 symptomatic patients (13.3%) had been completely asymptomatic before diagnosis, indicating that acute reactions may occur even in children without previous clinical manifestations of CeD.

### 3.3 Context of gluten exposure

The reported contamination incidents most frequently occurred in external dining settings, such as restaurants (46.2%), school cafeterias (26.9%), and during international travels (7.7%). Nonetheless, contamination events were not limited to external environments, as 19.2% of the cases occurred in the home setting. Further analysis of the types of foods implicated in these episodes revealed that contamination most often involved commonly consumed, high-risk items. In the majority of these episodes, the foods were believed by caregivers to be gluten-free at the time of consumption. Often, they were either purchased as labeled gluten-free products (e.g., certified gluten-free ice cream) or prepared in settings where caregivers had received assurances about gluten-free preparation, such as restaurants or social events. All of these food types were reported at similar frequencies (25%).

## 4 Discussion

This study highlights the high frequency of symptoms experienced by children with CeD on treatment with the GFD following unintentional gluten exposure, predominantly manifesting as gastrointestinal disturbances such as abdominal pain, diarrhea, and vomiting. These symptoms usually manifest abruptly, may be severe but tend to disappear spontaneously within hours or a few days. Interestingly they can present also in children who have been symptomless before starting treatment with the GFD. This finding suggests that acute reactions can occur regardless of pre-diagnosis symptom profile.

Our findings are consistent with other studies evaluating symptoms after gluten challenge reporting vomiting in 8%–44% and nausea in 13%–61% of the patients (15). A survey conducted by Silvester et al. (16) found a higher prevalence of symptomatic patients after suspected gluten exposure compared to the findings in the current study. This discrepancy may be attributed to population differences. Specifically, the adult population in Silvester's study may have experienced higher exposure rates due to less stringent supervision compared to the pediatric population in the current study, where parents or caregivers likely ensure greater dietary vigilance and reduce the likelihood of gluten contamination.

As expected, patients who were symptomatic at diagnosis and those diagnosed at an earlier age appeared more likely to experience symptoms following inadvertent gluten exposure. This may be due to the heightened mucosal sensitivity and immunological responses in those with more severe or long-standing disease.

The context of symptom onset is also of considerable importance. The high proportion of patients reporting symptoms when dining outside the home underscores the challenges faced by individuals with CeD in strictly adhering to a GFD (13, 14). This is particularly problematic in settings where they lack direct control over food preparation and ingredients. Interestingly, a study by Monzani et al. (17) found that one-third of survey respondents experienced improved adherence to the GFD during COVID lockdown measures, especially among those with previously poorer disease control. This suggests that the opportunity to avoid potential sources of gluten contamination and increased use of naturally gluten-free products contribute to better dietary adherence and symptom management in this patient population. Current methods for monitoring GFD adherence, such as dietary questionnaires, celiac serology, or clinical symptoms, are not sensitive enough to detect occasional dietary transgression (18). Novel non-invasive biomarkers such as gluten immunogenic peptides (GIP), while looking promising for assessing gluten ingestion, fall short of reliably capturing all meaningful exposures and comprehensively monitor adherence to a GFD (19).

While the reported symptoms may be attributable to factors other than gluten, such as FODMAPs, fructose or lactose intolerance, or the “nocebo” effect, a recent double-blind study found that patients with challenged with vital wheat gluten exhibited an elevated interleukin-2 response in 97% of participants, which correlated with the severity of nausea and vomiting, in contrast to a sham low-FODMAP challenge (20). This suggests that inadvertent gluten consumption is a key driver of the elevated immune response and associated symptoms in these individuals. Additionally, the rapid onset of symptoms within 2–3 h of gluten ingestion indicates that unintentional gluten exposure is the primary trigger for an heightened non-IgE immune response, not only in the chronic exposure scenario typical of the T cell-mediated condition, but also following an acute gluten challenge. A study by Tye-Din et al. (21) demonstrated that serum interleukin-2 levels, which were undetectable at baseline, became elevated within 4 h in 92% of patients with CeD following an acute gluten challenge. Additionally, the peak interleukin-2 concentration was correlated with the severity of symptoms, particularly nausea and vomiting. Other research has corroborated these findings, with most reactions occurring within 1 h of suspected gluten ingestion and resolving within 48 h (22). These findings are of major significance for understanding the pathophysiology of CeD, as they highlight the previously unappreciated importance of interleukin-2 together with interleukin-8 and interleukin-10 in driving the gluten-specific CD4+ T cell response responsible for the early immune events and clinical symptoms observed after gluten exposure (23). These recent insights into the acute immune response to gluten exposure highlight an evolving trend in CeD research. Traditionally, CeD has been considered a condition primarily driven by chronic immune activation. However, increasing evidence points to the presence of immediate, measurable immune responses following even minor gluten exposure, fundamentally shifting our understanding of symptom manifestation in patients with CeD (23). Future research should aim to further elucidate the mechanisms underlying these reactions and develop strategies to mitigate inadvertent gluten exposure.

A particularly noteworthy observation from our study is that 63% of symptomatic participants reported experiencing symptoms similar to those they had at the time of CeD diagnosis, while a distinct subset reported new symptom patterns following gluten re-exposure. This variation suggests a complex and individualized clinical response to gluten that may change over time. The recurrence of similar symptoms in the majority of cases likely reflects the reactivation of immune pathways previously involved in the initial disease presentation (24). However, the emergence of new symptoms in others—most commonly gastrointestinal—points to a dynamic interplay between immunological memory, dietary factors, and mucosal adaptation. One potential explanation is that ongoing low-level immune sensitization, despite mucosal healing on a strict GFD, may prime the gut for exaggerated responses upon re-exposure (25). Alternatively, shifts in gut microbiota composition, evolving dietary patterns, or partial recovery of intestinal barrier function may alter the symptomatic profile over time (26, 27). These evolving insights into the acute phase of gluten-induced symptoms have practical consequences for clinical care and research. They reinforce the concept that acute responses to gluten are multifaceted and patient-specific, which has important implications for clinical follow-up. It also highlights the need for individualized dietary counseling and symptom tracking, particularly for patients who develop novel symptoms post-diagnosis.

The retrospective design and reliance on self-reported data in this study may have led to potential recall bias. Additionally, the researchers did not independently confirm the reported instances of gluten contamination. However, the responses were validated by a dietitian, and strict criteria were used to determine a positive reaction. Furthermore, the relatively large sample size and real-world referral context help to mitigate these limitations, as this type of information is commonly encountered by clinicians during their interactions with patients. Most previous studies exploring symptoms in response to gluten exposure have focused on controlled gluten challenges, but we know that the specific type of grain consumed, and the food processing methods can also influence the resulting symptom profiles. Therefore, the variable nature of the inadvertent gluten exposures encountered in real life may yield symptom patterns that differ from those observed in the controlled challenge settings.

## 5 Conclusion

This study offers valuable insights into the frequency and nature of symptomatic responses experienced by children and adolescents with CeD following an acute gluten exposure. The findings in symptomatic patients highlight the high prevalence of gastrointestinal symptoms, including abdominal pain, diarrhea, and vomiting, within this patient population. The reactions are acute, usually occurring within 1–3 h after ingestion, with the most suspected settings of contamination being school cafeterias and dining outside the home. These results emphasize the critical need for continued research and development of effective tools to monitor and manage inadvertent gluten intake, in order to improve the quality of life of individuals living with CeD.

## Data Availability

The raw data supporting the conclusions of this article will be made available by the authors, without undue reservation.
